# The prevalence and correlates of the double burden of malnutrition among women in Ghana

**DOI:** 10.1371/journal.pone.0244362

**Published:** 2020-12-28

**Authors:** Sandra Boatemaa Kushitor, Lily Owusu, Mawuli Kobla Kushitor

**Affiliations:** 1 Food Security Initiative and Centre for Complex Systems in Transition, Stellenbosch University, Stellenbosch, South Africa; 2 Regional Institute for Population Studies, University of Ghana, Accra, Ghana; 3 Department of Health Policy, Planning and Management, University of Health and Allied Sciences, Hohoe, Ghana; African Population and Health Research Center, KENYA

## Abstract

Anaemia and underweight or overweight/obesity are major public health problems driving maternal and child mortality in low- and middle-income countries. While the burden of these conditions is recognised, the evidence for the co-occurrence of these conditions is fragmented and mixed, especially at the individual level. Further, many studies have focused on families and communities. The different pathways for the occurrence of anaemia and BMI challenges indicate that an individual can potentially live with both conditions and suffer the complications. This study examined the prevalence and factors associated with the co-occurrence of anaemia and BMI challenges among a cohort of women in Ghana. Data from the 2014 Ghana Demographic and Health Survey were used. The sample size was 4 337 women aged 15–49 years who were not pregnant during the survey. Women who suffered simultaneously from underweight or overweight/obesity and anaemia were considered as having the double burden of malnutrition. The data were analysed using descriptive statistics, Chi-square test and logistic regression in STATA. One-fifth of the participants were overweight (21%), 4% were underweight and about one-tenth were obese (12%). The prevalence of anaemia was 41%. Only one in three women had normal weight and was not anaemic (34%). About 14% of the women experienced the double burden of malnutrition. Being overweight and anaemic (57%) was the most common form of this double burden. Age, marital status, parity, and wealth were t key risk factors associated with the double burden of malnutrition. The findings from this study show that women experience multiple nutritional challenges concurrently and that only a few women had healthy nutritional status. This information is particularly important and can be introduced into health education programmes to help address misconceptions about body weight and health.

## Introduction

Several countries are facing a double burden of nutrition-related diseases, namely the coexistence of undernutrition or overnutrition and micronutrient deficiency in the same individual, household, community and population [1]. Anaemia is known to be one of the major micronutrient deficiencies [2]. Anaemia is associated with high maternal and infant mortality and reduced productivity [3, 4]. While the prevalence of stunting is decreasing significantly, overweight/obesity in all age groups is increasing [5]. The double burden of malnutrition causes great economic and health stress for governments, households and individuals [3]. Most countries are struggling with addressing the double burden of anaemia and underweight or overweight/obesity, as reported by the 2018 Global Nutrition Report [6].

Women have a higher burden of anaemia and underweight or overweight/obesity compared to men [7, 8]. Many women experience anaemia due to malaria, helminthic infection, pregnancy and nursing [9, 10]. Anaemia prevalence among women attending antenatal clinics ranged from 22% in Uganda [9] to 66% in the Dangme East area of Ghana [11]. In South Africa, 68% of women were overweight/obese compared to 41% of men [12]. This gender difference in malnutrition is further worsened by the effects of age, parity, wealth and urbanisation [13].

Previous studies have reported a strong correlation between body mass index (BMI) status and anaemia among women [14, 15]. While underweight is positively related to anaemia, the results of the relationship between overweight/obesity and anaemia are mixed. Among Indonesian and Ghanaian pregnant women, those with a high BMI were less likely to be anaemic [16], but in Mexico, obese women were more likely to be anaemic [17]. In Iran, there was no association between overweight/obesity and anaemia [18]. However, evidence of the association between BMI status and anaemia among non-pregnant women is non-existent in the Ghanaian context. Therefore, this study was conducted to examine the coexistence of anaemia and underweight or overweight/obesity in Ghana.

The prevalence of anaemia among women is high in Ghana [19]. In the Ashanti region, more than half of women are anaemic [20]. At the same time, overweight and obesity have been increasing in the country. Using the World Health Organization Study on AGEing and adult health (SAGE) data, it was estimated that about 20% and 10% were overweight and obese in 2007/08, respectively. In 2014/15, the proportion who were overweight and obese among this sample had increased to 24% and 16%, respectively [21].

However, research and intervention concerning anaemia and BMI challenges have been condition specific [22, 23]. Little attention has been given to the coexistence of these nutritional challenges among women, even though many women are reported to suffer from both anaemia and a BMI that is too low or high. Due to the preference for large body size in certain cultures, some women may be unaware of the micronutrient deficiencies that they may be suffering from [24]. This complex double burden underscores the importance of understanding how underweight or overweight/obesity and anaemia coexist among vulnerable populations. Also, the study of the double burden of malnutrition has primarily focused on the household level by examining the co-occurrence of overnutrition among mothers and undernutrition among their children [1]. This study examined the coexistence of anaemia and BMI challenges among women using the 2014 Ghana Demographic and Health Survey. The findings are essential for tracking efforts towards achieving the 2025 global nutrition targets.

## Methods

### Source of data

Cross-sectional data were retrieved from the 2014 Ghana Demographic and Health Survey, a nationally representative household survey of Ghana. The survey was carried out by the Ghana Statistical Service, the Ghana Health Service and the National Public Health Reference Laboratory of the Ghana Health Service. The protocol of the study, including the biomarker component, was reviewed and approved by the Ghana Health Service Ethical Review Committee and the Institutional Review Board of ICF International.

The 2014 Ghana Demographic and Health Survey used a two-stage sampling approach. The first stage selected 427 enumeration areas identified from the 2010 Ghana Population and Housing Census [25]. The households in the enumeration areas were listed. About 30 households were randomly selected from the enumeration areas. Field work for the survey was carried out from September to December 2014. The survey collected data on the fertility, socioeconomic status, health status and nutritional status of participating households and individuals using the Demographic and Health Survey Phase VI core questionnaire. All women aged 15–49 from the selected households were eligible to be interviewed. Data on anthropometry (weight and height) and haemoglobin were gathered for 4 528 women who consented. In this study, the sample was limited to 4 337 women who had complete data for all the variables of interest and were not pregnant at the time of the survey.

### Measurements

During the survey, blood samples were collected from women via finger prick. Haemoglobin concentration was analysed using a battery-operated portable HemoCue analyser. Participants with altitude-adjusted Hb < 120 g/dl were classified as anaemic, while those with Hb > 120 g/dl were classified as not anaemic. Besides, height and weight were measured. The BMI of the women was computed by dividing weight in kilograms by height in metres squared (kg/m^2^). Using the standard World Health Organization’s cut-off points, BMI categories were defined as underweight (< 18.5 kg/m^2^), normal weight (18.5–24.99.0 kg/m^2^), overweight (25.0–29.9 kg/m^2^) and obese (≥ 30.0 kg/m^2^) [26]. A dependent variable called nutritional status was created using anaemia and BMI status. Women who simultaneously were anaemic and underweight or overweight/obesity were considered as having the double burden of malnutrition. This included women who were 1) both anaemic and underweight; 2) both anaemic and overweight; and 3) both anaemic and obese. Women who were only 1) not anaemic; 2) underweight; 3) normal weight; 4) overweight; and 5) obese were classified as not having the double burden of malnutrition.

The covariates that were examined in this study included age, place of residence, educational attainment, ethnicity, marital status, parity, household wealth, breastfeeding status and fruit and vegetable intake. The age of respondents was categorised into 10-year age groups: 15–24, 25–34, 35–44 and 45–49 years. Place of residence was defined as urban or rural. Educational attainment was categorised as none (no formal education), primary, secondary and higher. Ethnicity was defined as Akan, Ga-Dangme, Ewe, Mole-Dagbani or other. Marital status was categorised as never married, currently married or formerly married. The parity of women was defined as the number of children ever borne. Household wealth was determined from household asset data using principal component analysis. The list of household assets used included household assets (such as television, bicycle, and iron) as well as characteristics of the dwelling place such as source of drinking water, sanitation facilities and type of flooring material. Household wealth was equally divided into five groups to create wealth quintiles, namely poorest, poor, middle, richer and richest. The number of days per week that participants had fruit and vegetables to eat was recorded as 0–7.

### Analysis

Descriptive statistics including means, frequencies and percentages were computed for all the variables. Cross-tabulations with Chi-square tests were used to show variation in nutritional status and the independent variables. To examine the independent effect of the covariates, we conducted a logistic regression. Odds ratios were calculated to examine the probability of experiencing the double burden of malnutrition, controlling for other factors. The odds ratios, confidence intervals and P-values were reported. The level of significance was set at P < 0.05. The data were weighted before analysis using the Demographic and Health Surveys women’s individual sample weights. The survey weight in STATA was set up using the generated weighting variable, primary sampling unit and strata. These were then applied in all the analyses. Multicollinearity was tested for using the variance inflation factor (VIF) and tolerance. The results of the VIF test are shown in S1 File. None of the VIF values is greater than 10, and the tolerance values are less than 0.1; therefore, there are no signs of multicollinearity. The analysis was conducted in STATA.

## Results

### Characteristics of respondents

A total of 4 337 women were involved in this study. The mean age was 30 ± 9.9 years, and about half of them (50%) lived in urban areas (Table 1). About half of them had a secondary school education (52%). The majority of the respondents were currently married (56%), and two thirds were Akan (44%). About one-fifth of the participants belonged to households in the richest wealth quintiles (17%). The number of children ever born ranged between 0 and 13 among the women (2.49±2.47).

**Table 1:** Sociodemographic characteristics of respondents.

Variable	Frequency	Percent
**Age**		
15–24	1 570	36.2
25–34	1 258	20.0
35–44	1 092	25.2
45–49	417	9.6
**Place of residence**		
Urban	2 138	49.3
Rural	2 199	50.7
**Level of education**		
No education	1 050	24.2
Primary	829	19.1
Secondary	2 240	51.7
Higher	218	5.0
**Marital status**		
Never married	1 447	33.4
Currently married	2 442	56.3
Formerly married	448	10.3
**Ethnicity**		
Akan	1 905	43.9
Ga-Dangme	238	5.5
Ewe	511	11.8
Mole-Dagbani	1 607	37.0
Other	76	1.8
**Wealth quintile**		
Poorest	1 105	25.5
Poorer	792	18.3
Middle	893	20.6
Richer	813	18.7
Richest	734	16.9
**Parity (mean)**	2.49 (± 2.47)	
**Fruit consumption, days per week (mean)**	3.45 (± 2.56)	
**Vegetable consumption, days per week (mean)**	3.42 (± 2.41)	
**Total**	4 337	100.0

### Nutritional status of respondents

**Fig 1:**
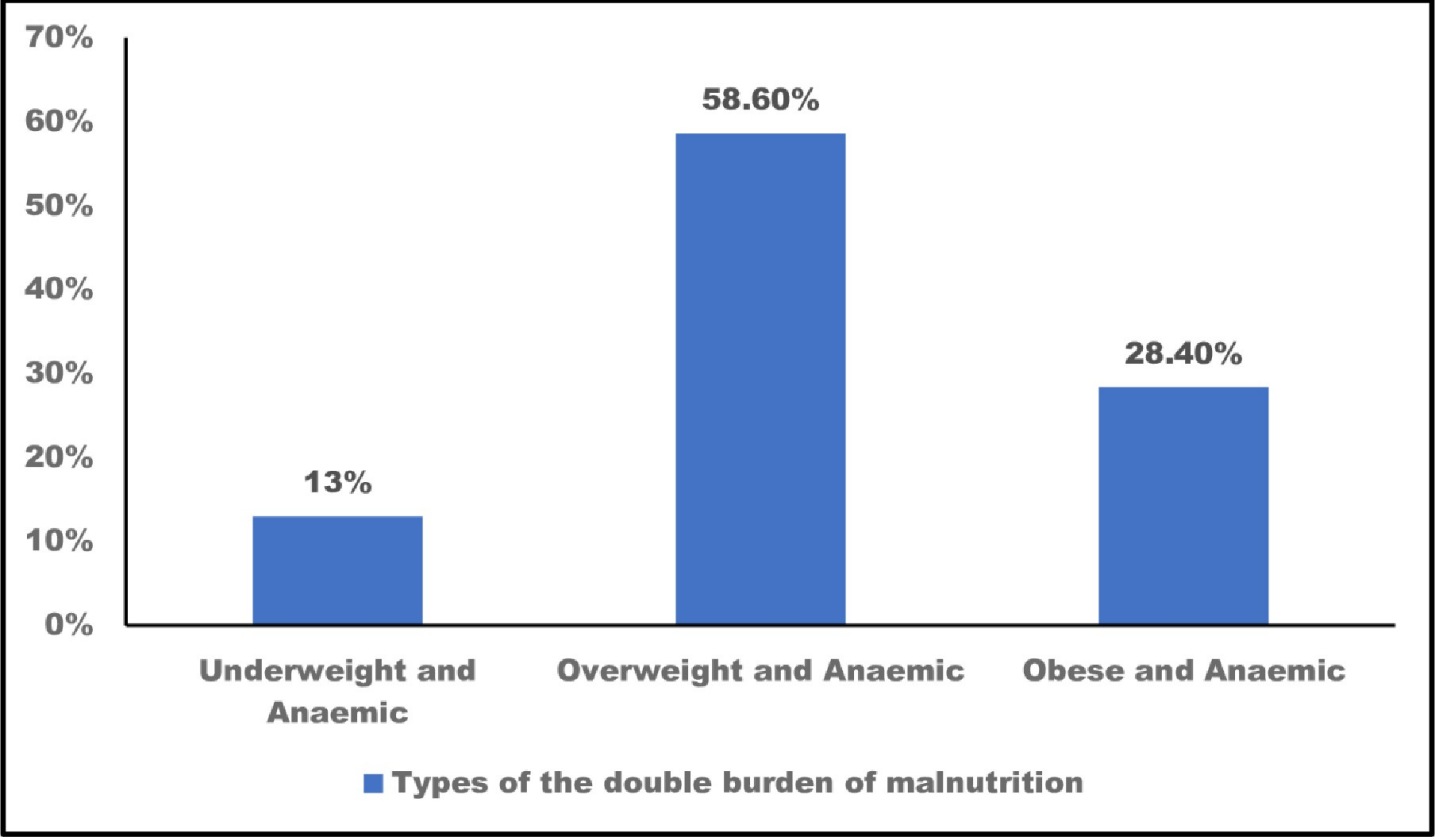
Types of the double burden of malnutrition among women in Ghana (n = 616).

The nutritional status of respondents is shown in Table 2. About two-fifths of the respondents were anaemic (41%). Based on the BMI classification, 61% were of normal weight and about 4% were underweight. About one in five of the women was overweight (23%), and 12% were obese. Only one in three women was normal weight and not anaemic (34%). Slightly more than 14% of the women had the double burden of malnutrition. As shown in Fig 1, being overweight and anaemic was the most common form of this double burden (59%),

**Table 2:** Nutritional status of respondents.

Nutritional status	Percent	Frequency
**Anaemia status**		
Anaemic	41.4	1 795
Not anaemic	58.6	2 542
**Body mass index**		
Underweight	3.6	158
Normal weight	61.1	2 650
Overweight	22.8	990
Obese	12.4	539
**Nutritional status**		
Double burden	14.2	616
No double burden	85.8	3 721
**Nutritional status (breakdown)**		
Underweight and not anaemic	1.8	78
Underweight and anaemic	1.8	80
Normal weight and not anaemic	33.9	1 471
Normal weight and anaemic	27.2	1 179
Overweight and not anaemic	14.5	629
Overweight and anaemic	8.3	361
Obese and not anaemic	8.4	364
Obese and anaemic	4.1	175
**Total**	**100**	**4 337**

### Distribution of nutritional status by background characteristics

Table 3 displays the percent distribution of the double burden of malnutrition according to background characteristics among women in Ghana. Particularly, older women showed a significantly higher prevalence of the double burden compared with younger individuals. For example, about 9% of the 15-24-year-old women showed no double burden compared to 19% of those aged 35–44 years (P = 0.000). The proportion of women who had the double burden was higher in urban areas (17%) than in rural areas (11%). Higher education did not have a protective effect on nutritional status. The proportion of women living with the double burden increased from 11% among those with no education to 17% among those with higher education. The double burden was higher among formerly married (22%) women than among those who had never married (9%) and those who were currently married (15%). Regarding wealth, women from poor households had a lower proportion of respondents with double burden (8%) than those from richer households (20%). The mean number of children ever born was higher among women with the double burden (2.8 ± 2.4) compared to their counterparts (2.4 ± 2.5). The mean number of days per week of fruit and vegetable consumption was similar among the women.

**Table 3:** Percent distribution of the double burden of malnutrition.

Variable	Double burden	No double burden	P-value
**Age**			
15–24	8.8	91.2	0.000
25–34	15.9	84.1	
35–44	19.3	80.7	
45–49	16.1	83.9	
**Place of residence**			0.000
Urban	17.1	82.9	
Rural	11.4	88.6	
**Level of education**			0.008
No education	11.0	88.9	
Primary	14.6	85.4	
Secondary	15.2	84.8	
Higher	17.4	82.6	
**Currently breastfeeding**			0.140
No	14.6	85.3	
Yes	12.8	87.2	
**Marital status**			0.000
Never married	9.2	90.8	
Currently married	15.6	84.4	
Formerly married	22.5	77.5	
**Ethnicity**			0.000
Akan	16.1	83.9	
Ga-Dangme	19.8	80.2	
Ewe	17.6	82.4	
Mole-Dagbani	9.8	90.2	
Other	18.4	81.6	
**Wealth quintile**			0.000
Poorest	7.6	92.4	
Poorer	13.4	86.6	
Middle	15.7	84.3	
Richer	16.7	83.3	
Richest	20.4	79.6	
**Parity**	2.1 ± 1.69	1.8 ± 1.69	0.000
**Fruit consumption, days per week**	3.4 ± 2.6	3.4 ± 2.6	0.400
**Vegetable consumption, days per week**	3.4 ± 2.40	3.4 ± 2.49	0.449

### Correlates of nutritional status

The correlates of the double burden of malnutrition are presented in Table 4. After controlling for other factors, the variables that were significantly related to nutritional status were age, marital status, wealth and parity. The odds of having the double burden of malnutrition was significantly higher for women aged 35–44 years compared to those aged 15–24 years (OR = 1.54; 95% CI = 1.09, 2.12). Compared with women who had never married, women who were formerly married were twice as likely to have the double burden of malnutrition (OR = 2.03; 95% CI = 1.37, 3.01). Household wealth was associated with the double burden of malnutrition–women who belonged to households of the middle (OR = 2.10; 95% CI = 1.44, 3.06), richer (OR = 2.14, 95% CI = 1.38, 3.32) and richest (OR = 2.75; 95% CI = 1.73–4.37) wealth quintiles had significantly higher odds of experiencing the double burden of malnutrition. Every additional birth also increased the odds of the double burden of malnutrition (OR = 1.06; 95% CI = 1.00–1.12).

**Table 4:** Correlates of the double burden of malnutrition among women in Ghana.

Variable	Odds ratio	95% Confidence interval	P-value
**Age**			
15–24 (ref)			
25–34	1.39	1.05–1.86	0.022
35–44	1.54	1.09–2.12	0.013
45–49	1.13	0.71–1.82	0.597
**Place of residence**			
Urban (ref)			
Rural	1.01	0.84–1.43	0.497
**Level of education**			
No education (ref)			
Primary	1.14	0.85–1.53	0.367
Secondary	1.20	0.92–1.56	0.179
Higher	1.18	0.73–1.91	0.488
**Currently breastfeeding**			
No (ref)			
Yes	0.89	0.70–1.15	0.393
**Marital status**			
Never married (ref)			
Currently married	1.42	1.05–1.92	0.020
Formerly married	2.03	1.37–3.01	0.000
**Ethnicity**			
Akan (ref)			
Ga-Dangme	1.32	0.95–1.84	0.900
Ewe	1.30	0.97–1.75	0.075
Mole-Dagbani	0.93	0.71–1.22	0.616
Other	1.20	0.59–2.44	0.595
**Wealth quintile**			
Poorest (ref)			
Poorer	1.72	1.26–2.35	0.001
Middle	2.10	1.44–3.06	0.000
Richer	2.14	1.38–3.32	0.001
Richest	2.75	1.73–4.37	0.000
**Parity**	1.06	1.00–1.12	0.045
**Fruit consumption, days**	0.99	0.95–1.03	0.793
**Vegetable consumption, days**	0.99	0.96–1.04	0.959

ref: Reference group.

## Discussion

The double burden of anaemia and underweight or overweight/obesity affects women disproportionately. However, research interventions have not focused on the co-occurrence of anaemia and BMI challenges among women. This study examined the co-occurrence of anaemia and BMI challenges and the associated risk factors among women in Ghana. The overall prevalence of anaemia was 41% and that of obesity was 12%. Only 34% of women had normal weight and were not anaemic. The findings indicate that 14% of women had the double burden of malnutrition. Among these women, being overweight and anaemic was the most common situation. The prevalence of the double burden of malnutrition was strongly correlated with age, marital status, parity and wealth.

It has been reported that the prevalence of the double burden of malnutrition at the household level is high in low- and middle-income countries, especially in sub-Saharan Africa [5]. The prevalence of the within-person double burden of underweight or overweight/obesity and anaemia found in this study (14%) was lower than what had been estimated for Kenya (19%) [27] and India (23%) [28]. Even though the prevalence of the individual-level double burden reported in this study is relatively low, about half of the women were overweight and anaemic. This indicates that a high proportion of women are experiencing both undernutrition and overnutrition simultaneously.

In studies that focused on predicting either obesity or anaemia, age, marital status, wealth and parity have been reported as risk factors [12, 20, 29]. These variables are biological and demographic risk factors as well as indicators of socioeconomic status. Age and parity are leading risk factors of maternal malnutrition. Pregnancy and the postpartum period result in substantial demands for iron, which accounts for the biological vulnerability of women to anaemia. For example, during pregnancy, enlargement of red-cell mass and the development and maintenance of the placenta increase iron requirements substantially from 0.8 mg per day in the first trimester to 7.5 mg per day in the third trimester [30]. At the same time, pregnancy and postpartum food cultures increase the risk of obesity among women. Wealthy individuals have access to a wider diversity of nutrient-limited foods and report high intake of fat-rich and sugar-rich diets. In Nigeria, women from rich households consume fast foods at least twice a week [31].

Furthermore, weight management practices among women can explain the results of the study. Weight loss is one of the negative effects of anaemia [32]. For cultural reasons, women often attach great importance to appearance and are preoccupied with their weight at a very early age. In some cultures, the plump woman is idealised, as it is perceived to be preferred by men [33]. In African countries, including Ghana, a plump woman is considered as wealthy and happy [24, 34]. As a result, weight gain has minimal negative connotations and is a cultural solution to anaemia [35, 36]. In an attempt to treat anaemia, the majority of women resort to weight management practices such as intake of appetite-stimulating supplements that predispose them to overweight and obesity [37]. However, gaining additional body fat may prevent iron homeostasis and increase the risk of anaemia. It is reported that obese individuals have an impaired ability to absorb iron. Among women in Chile, it was reported that obese women (20%) had lower iron absorption levels compared to normal-weight women (33%) [15].

To the best of our knowledge, this study is the first of its kind to provide empirical evidence of the within-person double burden of malnutrition with nationally representative data from Ghana. The findings also highlight the risk factors that predispose women to the double burden of malnutrition. Although this study used a nationally representative dataset and controlled for other factors, the findings should be interpreted in the light of its limitations. Firstly, this study was cross-sectional and collected data at a single time point. We cannot make a causal claim about the observed associations between anaemia and underweight or overweight/obesity and the concomitant risk factors. Secondly, detailed dietary consumption data were not collected for women during the 2014 survey as was the case in the 2008 survey [38, 39]. The only available information was the number of days per week of consuming vegetables and fruit; the type and quantity of fruit and vegetables consumed were not measured. We recommend that future versions of the Ghana Demographic and Health Survey include detailed questions on fruit and vegetable intake among women.

## Conclusion

There is a gap in research on the prevalence of and the factors that influence the within-person double burden of malnutrition among African women. Our study suggests that Ghanaian women simultaneously experience underweight or overweight/obesity and anaemia and that only a few have a healthy nutritional status. The co-occurrence of overweight and anaemia was the most common form of the double burden of malnutrition. Age, marital status, parity and wealth were the key risk factors associated with the double burden of malnutrition. The pathways through which these risk factors influence the double burden of malnutrition are unhealthy lifestyles, especially poor diet, physical inactivity and inadequate weight management behaviours. These factors are potentially modifiable through educational efforts targeting women and social norms. Future research should focus on addressing beliefs about body weight and anaemia. Such research can develop culturally sensitive educational content on pregnancy and breastfeeding food beliefs. Also, these interventions should be delivered at the individual and community level. Lastly, such research can investigate the impact of these efforts on body weight and anaemia outcomes.

## Supporting information

S1 File (DOCX)
