# Supplementary material for: The prevalence and correlates of the double burden of malnutrition among women in Ghana
Source: PLoS One. 2020 Dec 28;15(12):e0244362. doi: 10.1371/journal.pone.0244362 (PMC7769247; doi:10.1371/journal.pone.0244362)
Supplement: S1 File — (DOCX) [file pone.0244362.s001.docx]

**The double burden of anaemia, underweight and overweight/obesity among women in Ghana**

**Sandra Boatemaa Kushitor^1^*, Lily Owusu^2^, Mawuli Kobla Kushitor^3^**

^1^Food Security Initiative and Centre for Complex Systems in Transition, Stellenbosch University, Private Bag X1 Matieland 7602, Stellenbosch, South Africa. Email: Boatemaa@sun.ac.za

^2^Regional Institute for Population Studies, University of Ghana, Accra, Ghana. Email: [lowusu33@yahoo.com](mailto:lowusu33@yahoo.com)

^3^Department of Health Policy, Planning and Management, University of Health and Allied Sciences, Hohoe, Ghana. Email: [mkushitor@uhas.edu.gh](mailto:mkushitor@uhas.edu.gh)

***Corresponding author**

Sandra Boatemaa

Food Security Initiative and Centre for Complex Systems in Transitions, Stellenbosch University, Private Bag X1 Matieland 7602, Stellenbosch, South Africa.

Email: [boatemaa@sun.ac.za](mailto:boatemaa@sun.ac.za)

Table 1. **Multicollinearity tests.**

| **Variable** | **VIF** | **1/VIF** |
| --- | --- | --- |
| **Age** |  |  |
| 25-34 | 3.04 | 0.328458 |
| 35-44 | 4.32 | 0.231576 |
| 45-49 | 2.78 | 0.359876 |
| **Parity** | 6.04 | 0.16548 |
| **Fruit consumption, days** | 2.97 | 0.336438 |
| **Vegetable consumption** | 3.09 | 0.323773 |
| **Place of residence** |  |  |
| Urban | 3.77 | 0.265173 |
| **Level of education** |  |  |
| Primary | 1.7 | 0.589438 |
| Secondary | 3.69 | 0.271131 |
| Higher | 1.52 | 0.6574 |
| **Breastfeeding status** |  |  |
| Currently breastfeeding | 1.86 | 0.538923 |
| **Marital status** |  |  |
| Currently married | 6.39 | 0.156606 |
| Previously married | 1.97 | 0.507569 |
| **Ethnicity** |  |  |
| Ga-Dangme | 1.12 | 0.89631 |
| Ewe | 1.23 | 0.80976 |
| Mole-Dagbani | 1.82 | 0.549021 |
| Other | 1.04 | 0.961601 |
| wealth |  |  |
| Poorer | 1.77 | 0.564697 |
| Middle | 2.44 | 0.409511 |
| Richer | 3.09 | 0.323931 |
| Richest | 3.61 | 0.277039 |
